# Comparison between the Roche Cobas 4800 Human Papillomavirus (HPV), Abbott RealTime High-Risk HPV, Seegene Anyplex II HPV28, and Novel Seegene Allplex HPV28 Assays for High-Risk HPV Detection and Genotyping in Mocked Self-Samples

**DOI:** 10.1128/spectrum.00081-23

**Published:** 2023-06-07

**Authors:** Margo Bell, Bo Verberckmoes, Janne Devolder, Heleen Vermandere, Olivier Degomme, Yasmin Medeiros Guimarães, Luani Rezende Godoy, Elena Ambrosino, Piet Cools, Elizaveta Padalko

**Affiliations:** a International Center for Reproductive Health, Department of Public Health and Primary Care, Faculty of Medicine and Health Sciences, Ghent University, Ghent, Belgium; b Department of Diagnostic Sciences, Faculty of Medicine and Health Sciences, Ghent University, Ghent, Belgium; c Department of Obstetrics and Gynecology, Ghent University Hospital, Ghent, Belgium; d Molecular Oncology Research Center, Barretos Cancer Hospital, São Paulo, Brazil; e Institute for Public Health Genomics, Department of Genetics and Cell Biology, Faculty of Health Medicine and Life Sciences, Maastricht University, Maastricht, the Netherlands; f Department of Medical Microbiology, Ghent University Hospital, Ghent, Belgium; University of Utah and ARUP Laboratories

**Keywords:** analytical performance, HPV genotyping, human papillomavirus, molecular diagnostics

## Abstract

Infection with high-risk human papillomavirus (hrHPV) is well recognized as the main cause of cervical cancer. The recently developed Seegene Allplex HPV28 assay is a novel quantitative PCR (qPCR) assay designed to separately detect and quantify 28 distinct HPV genotypes in a fully automated and user-friendly manner. This study evaluated and compared the performance of this new assay with the performance of the Roche Cobas 4800, the Abbott RealTime high-risk HPV, and the Seegene Anyplex II HPV28 assays. A total of 114 mocked self-samples, i.e., semicervical samples collected by gynecologists using the Viba-Brush, were analyzed with all four HPV assays. Agreement in terms of detecting and genotyping HPV was assessed by the mean of the Cohen’s kappa (κ) coefficient. Results of all four HPV assays agreed in 85.9% of the cases when using the Abbott RealTime manufacturer’s recommended quantification cycle (*C_q_*) cutoff for positivity (<32.00) and 91.2% when using an adapted range (32.00 to 36.00). An intercomparison of the included assays demonstrated an overall agreement ranging from 85.9 to 100.0% (κ = 0.42 to 1.00) when using the manufacturer’s guidelines and 92.9 to 100.0% (κ = 0.60 to 1.00) with the adapted range. For all assays, highly significant, strongly positive Pearson correlations were shown between the *C_q_* values of positive test results. This study thereby shows high concordance between results of the included HPV assays on mocked self-samples. Based on these findings, we imply that the novel Allplex HPV28 assay demonstrates a comparable performance to those of available qPCR HPV assays, potentially providing opportunities for the simplification and standardization of future large-scale testing.

**IMPORTANCE** This study proves that the novel Allplex HPV28 assay has a good diagnostic performance in comparison with the well-known, validated, and frequently used Roche Cobas 4800, Abbott RealTime, and Anyplex II HPV28 assays. According to our experience, the novel Allplex HPV28 assay had a user-friendly and automated workflow with short hands-on time, had an open platform which facilitates the use of add-on assays, and provided quick and easy-to-interpret results. Together with its ability to detect and quantify 28 HPV genotypes, the Allplex HPV28 assay could therefore potentially provide opportunities for the simplification and standardization of future diagnostic testing programs.

## INTRODUCTION

Cervical cancer is the fourth most prevalent malignancy in women worldwide, with over 604,000 new cases and 342,000 deaths in the year 2020 (https://gco.iarc.fr/today/home). As of today, it has been proven that most of these cervical malignancies are associated with an infection by the human papillomavirus (HPV), the most common sexually transmitted pathogen worldwide (1). Accordingly, evidence shows that HPV is implicated in approximately 99% of cervical squamous cell cases and 89% of cervical adenocarcinoma cases (2). HPV is a small, nonenveloped DNA virus, possessing the ability to infect cutaneous or mucosal surfaces (3). More than 100 different strains of the virus have been uncovered, including roughly 30 to 40 genotypes that are able to infect the human genital tract (4). Recently, the International Agency for Research on Cancer (IARC) specified 14 high-risk HPV (hrHPV) genotypes, as follows: HPV16, 18, 31, 33, 35, 39, 45, 51, 52, 56, 58, 59, 66, and 68. HPV16 and 18 are considered the most oncogenic ones and account for over 70% of all worldwide cervical cancer cases (4).

Cervical cancer is considered a highly preventable disease, as it has been suggested that up to 93% of all cases could be prevented via HPV vaccination and/or cervical screening for HPV infection and (pre-)cancerous lesions (5). To accomplish regular screening, cervical cytology methods, i.e., methods detecting morphological changes in cervical cells, have been the standard of care for over 50 years in the majority of high-income countries (6). Through the Papanicolaou (PAP) smear and liquid-based cytology (LBC), we are able to visually distinguish normal cytology from low-grade squamous intraepithelial lesions (LSILs), high-grade squamous intraepithelial lesions (HSILs), atypical squamous cells with undetermined significance (ASC-US), and atypical squamous cells where HSILs cannot be excluded (ASC-H). However, since 2015, the European Guidelines for Quality Assurance in Cervical Cancer Screening encourage the use of HPV DNA tests for primary screening (7, 8). Not only have these methods proven to be more sensitive and efficient than cytology methods, they are also more objective as they do not rely on visual inspection expertise (6).

Over the past years, over 100 different HPV assays have been developed, and they widely vary in the number and type of target HPV genotypes, their working mechanism, and their clinical utility (9, 10). Among these DNA tests, multiplex quantitative PCR (qPCR) assays are currently widely used for both detecting and genotyping HPV. These assays are high throughput and overall feature a low risk of cross-contamination of PCR products (11). Two well-known qPCR HPV assays are the Abbott RealTime HR HPV assay and the Roche Cobas 4800 HPV assay. Both assays have been approved for clinical purposes by the U.S. Food and Drug Administration (FDA), and multiple studies have already compared their performances (12–14). Recently, Seegene also developed two novel qPCR HPV assays, including the Anyplex II HPV28 and Allplex HPV28 detection assays. Both assays are based on the unique tagging oligonucleotide cleavage and extension technology (TOCE), which allows for the detection of multiple HPV targets in a single fluorescence channel on qPCR instruments. The Anyplex II HPV28 assay utilizes the TOCE system in combination with a cyclic melting temperature analysis and generates semiquantitative results (15, 16), whereas the more recently developed Allplex HPV 28 assay uses a multiple detection temperature technology, generating individual quantification cycle (*C_q_*) values for 28 distinct genotypes at once.

Interestingly, considering the novelty of the Allplex 28 HPV assay, no published studies have compared this assay with commercially available HPV assays. Therefore, in this study, we compared the diagnostic performance of the Allplex HPV28, the Anyplex II HPV28, the Abbott RealTime HR HPV, and the Roche Cobas 4800 HPV assay for their ability to detect and genotype hrHPV in mocked self-samples. In Table 1, an overview of the characteristics of the four included HPV assays is summarized.

**TABLE 1:** Characteristics of the four included qPCR HPV assays^*a*^

qPCR HPV assay	Type of technology	Gene target	HPV target	*C_q_* cutoff target positivity^*b*^	Internal quality control
Abbott RealTime HR	Target amplification (qPCR)	L1 gene	HPV16 and 18, pool of 12 other hr genotypes^*c*^	All HPV types: 32.00	Human β-globin gene
Roche Cobas	Target amplification (qPCR)	L1 gene	HPV16 and 18, pool of 12 other hr genotypes^*c*^	HPV16: 40.5, HPV18 and others: 40.0	Human β-globin gene
Anyplex II HPV28^*d*^	Target amplification (high multiplex, TOCE)	L1 gene	HPV6, 11, 16, 18, 26, 31, 33, 35, 39, 40, 42, 43, 44, 45, 51, 52, 53, 54, 56, 58, 59, 61, 66, 68, 69, 70, 73, 82	NA	Human β-globin gene
Allplex HPV28^*d*^	Target amplification (high multiplex, TOCE)	L1 gene	HPV6, 11, 16, 18, 26, 31, 33, 35, 39, 40, 42, 43, 44, 45, 51, 52, 53, 54, 56, 58, 59, 61, 66, 68, 69, 70, 73, 82	All HPV types: 42.00	Human β-globin gene

a *C_q_*, cycle threshold; hr, high-risk; NA, not applicable.

b As recommended by the manufacturer.

c Combined results of other hrHPV genotypes include HPV31, 33, 35, 39, 45, 51, 52, 56, 58, 59, 66, and 68.

d Study includes only results of the 14 hrHPV genotypes.

## RESULTS

### Clinical and sociodemographic description of the study population.

An overview of the clinical and sociodemographic characteristics of the study population is shown in Table 2. A total of 24 participants (21%) were recruited in Belgium, 29 (25%) in Brazil, 41 (36%) in Ecuador, and 20 (18%) in Portugal. The average age of participants was 45 years, with 3 (3%) of the participants aged under 30, 49 (43%) aged between 30 to 39, 28 (25%) aged between 40 to 49, and 34 (30%) aged over 50 years. Most participants were single (*n* = 33, 29%) or married (*n* = 32, 29%), 19 (17%) were living together or in a relationship, 18 (16%) were divorced, and 10 (9%) were widowed. One-third of women (*n* = 37, 33%) had a history of smoking, 20 (18%) were smoking during the study, and 75 (67%) had taken hormonal contraceptives for a period of over 5 years. Most women (*n* = 30, 26%) were diagnosed with normal cytology, 20 (18%) with LSILs, 26 (23%) with HSILs, 11 (10%) with ASC-US, 17 (15%) with ASC-H, and 10 (9%) with cervical cancer.

**TABLE 2:** Summary of clinical and sociodemographic characteristics of the study population

Characteristic (*n* = 114)	Count (*n*)	Percentage (%)
Country		
Belgium	24	21
Brazil	29	25
Ecuador	41	36
Portugal	20	18
Ethnogeographic origin other than country		
Yes	10	9
No	104	91
Age category		
<30 yrs	3	3
30–40 yrs	49	43
41–50 yrs	28	25
>51 yrs	34	30
Civil status^*a*^		
Single	33	29
Living together/in a relationship	19	17
Married	32	29
Divorced	18	16
Widow	10	9
Smoking history^*a*^		
Yes	37	33
No	75	67
Currently smoking^*a*^		
Yes	20	18
No	92	82
>5 yrs of hormonal contraceptives^*a*^		
Yes	75	67
No	37	33
Cervical cytology category^*b*^		
Normal	30	26
LSIL	20	18
HSIL	26	23
ASC-US	11	10
ASC-H	17	15
Cervical cancer	10	9

a Two missing values.

b ASC-H, atypical squamous cells where HSIL cannot be excluded; ASC-US, atypical squamous cells of undetermined significance; HSIL, high-grade squamous intraepithelial lesion; LSIL, low-grade squamous intraepithelial lesion.

### Detection of HPV (co)infections by the different HPV assays stratified by cervical cytology.

In Table 3, HPV cases detected by the different HPV assays are shown stratified by cervical cytology. While all assays were able to detect HPV DNA in 100% of the cancer cytology samples, no other cervical cytology category had a 100% positivity rate for all HPV assays. For all HPV assays, except for the Roche Cobas assay, positivity rates were higher for HSIL samples than those for LSIL samples. The Roche Cobas assay overall showed the highest positivity rate, with 100% HPV positivity for all cytology categories, apart from normal and ASC-US cytology. Coinfections were detected in 4.7 to 6.9% of all samples, with the Anyplex II HPV28 assay detecting the highest number of coinfections compared with other HPV assays (Table 3).

**TABLE 3:** Detection of HPV (co)infections by the different qPCR HPV assays stratified by cervical cytology results

Cytology (*n*)^*a*^	No. (positivity rate [%]) of HPV cases by assay				No. (coinfection rate [%]) of HPV coinfections^*b*^ by assay			
	Cobas	Abbott	Anyplex 28^*c*^	Allplex 28^*c*^	Cobas	Abbott	Anyplex 28^*c*^	Allplex 28^*c*^
Normal (30)	23 (76.7)	15 (50.0)	20 (66.7)	20 (66.7)	1 (4.3)	1 (6.7)	1 (5.0)	1 (5.0)
ASC-US (11)	10 (90.9)	9 (81.8)	10 (90.9)	10 (90.9)	1 (10.0)	1(11.1)	2 (20.0)	1 (10.0)
LSIL (20)	20 (100.0)	18 (90.0)	19 (95.0)	19 (95.0)	0 (0.0)	0 (0.0)	0 (0.0)	0 (0.0)
ASC-H (17)	17 (100.0)	14 (82.4)	16 (94.1)	16 (94.1)	1 (5.9)	1(7.1)	1 (6.3)	1 (6.3)
HSIL (26)	26 (100.0)	24 (92.3)	26 (100.0)	26 (100.0)	2 (7.7)	2 (8.3)	3 (11.5)	3 (11.5)
Cancer (10)	10 (100.0)	10 (100.0)	10 (100.0)	10 (100.0)	0 (0.0)	0 (0.0)	0 (0.0)	0 (0.0)
All (114)	106 (92.9)	90 (78.9)	101 (88.6)	101 (88.6)	5 (4.7)	5 (5.6)	7 (6.9)	6 (5.9)

a ASC-H, atypical squamous cells where HSIL cannot be excluded; ASC-US, atypical squamous cells of undetermined significance; HSIL, high-grade squamous intraepithelial lesion; LSIL, low-grade squamous intraepithelial lesion.

b Samples were considered coinfected when they were infected with both HPV16 and/or HPV18 and another hrHPV genotype.

c Study includes only results of the 14 hrHPV genotypes.

### Concordance for detecting any hrHPV genotype between the four HPV assays.

Table 4 and 5 document the concordance between the included HPV assays. When applying the Abbott manufacturer’s cutoff for positivity (Table 4), the test results agreed in 85.9% of all samples. Of these samples, 7.0% were diagnosed as HPV negative, while 78.9% were diagnosed as HPV positive. Among the samples found positive with all HPV assays, 12 samples were positive for HPV16, 7 samples were positive for HPV18, and 71 samples tested positive for other hrHPV genotypes. Moreover, 5 (4.4%) samples tested positive by the Roche Cobas HPV assay but negative with the other HPV assays and 11 (9.7%) samples were positive by all HPV assays, except for the Abbott RealTime HR HPV assay. After the use of the less stringent Abbott RealTime *C_q_* cutoff for positivity (Table 5), seven samples that were reported as negative with the manufacturer’s cutoff nevertheless demonstrated presence of hrHPV (*C_q_* value range, 32.32 to 35.52; median *C_q_*, 33.63). The HPV assays hereafter agreed in 91.2%, with 7.0% of all cases testing negative and 84.2% testing positive (Table 5). All samples that were additionally found positive contained hrHPV genotypes other than HPV16 and HPV18. Moreover, by using this less stringent Abbott RealTime cutoff, five samples still tested negative for HPV DNA by the Abbott RealTime assay while testing positive with all other HPV assays (*n* = 1, LSIL; *n* = 3, normal cytology) or with the Roche Cobas assay only (*n* = 1, LSIL).

**TABLE 4:** Concordance/discordance between the different qPCR HPV assays for any detectable HPV genotype using the Abbott RealTime manufacturer’s *C_q_* cutoff for positivity^*a*^

Result by assay				*n* (%) (total = 114)	Concordance (HPV genotype [*n*])^*d*^
Roche Cobas	Abbott RealTime HR	Anyplex 28^*b*^	Allplex 28^*b*^		
−	−	−	−	8 (7.0)^*c*^	NA
+	+	+	+	90 (78.9)^*c*^	HPV16 (12), HPV18 (7), Other hrHPV (71)
+	−	−	−	5 (4.4)	Other hrHPV (5)
+	−	+	+	11 (9.7)	Other hrHPV (11)

a The cut-off value is <32.00. All +/− combinations others than the ones presented did not prevail within this sample selection. No discrepant results were observed for HPV16 or HPV18 between the different assays.

b Study includes only results of the 14 hrHPV genotypes.

c Total agreement results were 98 (85.9%).

d hr, high-risk; NA, not applicable. Other hrHPV genotypes include HPV31, 33, 35, 39, 45, 51, 52, 56, 58, 59, 66, and 68.

**TABLE 5:** Concordance/discordance between the different qPCR HPV assays for any detectable HPV genotype using a less stringent Abbott RealTime *C_q_* range for positivity^*a*^

Result by assay				*n* (%) (total = 114)	Concordance (HPV genotype [*n*])^*d*^
Roche Cobas	Abbott RealTime HR	Anyplex 28^*b*^	Allplex 28^*b*^		
−	−	−	−	8 (7.0)^*c*^	NA
+	+	+	+	96 (84.2)^*c*^	HPV16 (12), HPV18 (7), Other hrHPV (77)
+	−	−	−	5 (4.4)	Other hrHPV (5)
+	−	+	+	4 (3.5)	Other hrHPV (4)
+	+	−	−	1 (0.9)	Other hrHPV (1)

a The cut-off range is 32.00–36.00. All +/− combinations others than the ones presented did not prevail within this sample selection. No discrepant results were observed for HPV16 or HPV18 between the different assays.

b Study includes only results of the 14 hrHPV genotypes.

c Total agreement results were 104 (91.2%).

d hr, high-risk; NA, not applicable. Other hrHPV genotypes include HPV31, 33, 35, 39, 45, 51, 52, 56, 58, 59, 66, and 68.

In Tables 6 and 7, the positive, negative, and kappa agreements between the four HPV assays are presented. Results are shown for the Abbott RealTime manufacturer’s *C_q_* cutoff for positivity (<32.00) (Table 6) and the less stringent Abbott RealTime *C_q_* cutoff range for positivity (32.00 to 36.00) (Table 7). The overall agreement initially ranged from 85.9% to 100.0%, with corresponding kappa coefficients ranging from 0.42 to 1.00 (Table 6). When using the less stringent Abbott RealTime *C_q_* range for positivity, however, these agreements ranged from 92.9% to 100.0%, with corresponding kappa coefficients ranging from 0.60 to 1.00 (Table 7). While for both the manufacturer-recommended and adapted conditions, the lowest agreement was seen between the Roche Cobas and Abbott RealTime HPV assays (85.9%, κ = 0.42; and 92.9%, κ = 0.60, respectively), the highest agreement was seen between the Anyplex and Allplex HPV28 assays (100%, κ = 1.00). Although the Anyplex and Allplex HPV28 assays demonstrate a perfect agreement in the examination of the results for the “other” hrHPV genotypes, discrepancies within these other hrHPV genotypes could be observed. Discordances were present for hrHPV genotypes 51, 52, and 59, with the Anyplex II HPV28 assay detecting 9, 13, and 10 samples, respectively, and the Allplex HPV28 assay detecting 10, 14, and 12 samples, respectively (see Table S1 in the supplemental material).

**TABLE 6:** Positive, negative, and overall kappa agreements between the results of the four HPV assays using the Abbott RealTime manufacturer’s *C_q_* cutoff for positivity^*a*^

Assay	Parameter	Results by assay		
		Abbott RealTime	Anyplex 28^*b*^	Allplex 28^*b*^
Roche Cobas	Overall agreement (95% CI^*c*^)	85.9 (76.64–91.11)	95.6 (89.34–98.45)	95.6 (89.34–98.45)
	Negative agreement (95% CI)	100.0 (63.06–100.00)	100.0 (63.06–100.00)	100.0 (63.06–100.00)
	Positive agreement (95% CI)	84.9 (78.21–91.76)	95.3 (89.34–98.45)	95.3 (89.34–98.45)
	Kappa coefficient (95% CI)	0.42 (0.21–0.63)	0.74 (0.52–0.95)	0.74 (0.52–0.95)
Abbott RealTime	Overall agreement (95% CI)		90.4 (83.39–95.08)	90.4 (83.39–95.08)
	Negative agreement (95% CI)		100.0 (75.29–100.00)	100.0 (75.29–100.00)
	Positive agreement (95% CI)		89.1 (81.35–94.44)	89.1 (81.35–94.44)
	Kappa coefficient (95% CI)		0.65 (0.47–0.84)	0.65 (0.47–0.84)
Anyplex 28^*b*^	Overall agreement (95% CI)			100.0 (96.41–100.00)
	Negative agreement (95% CI)			100.0 (75.29–100.00)
	Positive agreement (95% CI)			100.0 (96.82–100.00)
	Kappa coefficient (95% CI)			1.00 (1.00–1.00)

a The cut-off value is <32.00.

b Study includes only results of the 14 hrHPV genotypes.

c CI, confidence interval.

**TABLE 7:** Positive, negative, and overall kappa agreements between the results of the four HPV assays using a less stringent Abbott RealTime *C_q_* cutoff range for positivity^*a*^

Assay	Parameter	Results by assay		
		Abbott RealTime	Anyplex 28^*b*^	Allplex 28^*b*^
Roche Cobas	Overall agreement (95% CI)	92.9 (86.64–96.92)	95.6 (89.34–98.45)	95.6 (89.34–98.45)
	Negative agreement (95% CI)	100.0 (63.06–100.00)	100.0 (63.06–100.00)	100.0 (63.06–100.00)
	Positive agreement (95% CI)	92.4 (85.67–96.69)	95.3 (89.34–98.45)	95.3 (89.34–98.45)
	Kappa coefficient (95% CI^*c*^)	0.60 (0.37–0.83)	0.74 (0.52–0.95)	0.74 (0.52–0.95)
Abbott RealTime	Overall agreement (95% CI)		96.5 (91.26–99.04)	96.5 (91.26–99.04)
	Negative agreement (95% CI)		100.0 (76.84–100.00)	100.0 (76.84–100.00)
	Positive agreement (95% CI)		96.0 (90.07–98.90)	96.0 (90.07–98.90)
	Kappa coefficient (95% CI)		0.85 (0.70–0.99)	0.85 (0.70–0.99)
Anyplex 28^*b*^	Overall agreement (95% CI)			100.0 (96.41–100.00)
	Negative agreement (95% CI)			100.0 (75.29–100.00)
	Positive agreement (95% CI)			100.0 (96.82–100.00)
	Kappa coefficient (95% CI)			1.00 (1.00–1.00)

a The cut-off value is 32.00–36.00.

b Study includes only results of the 14 hrHPV genotypes.

c CI, confidence interval.

### Pearson correlation between *C_q_* values of the HPV assays.

**FIG 1:**
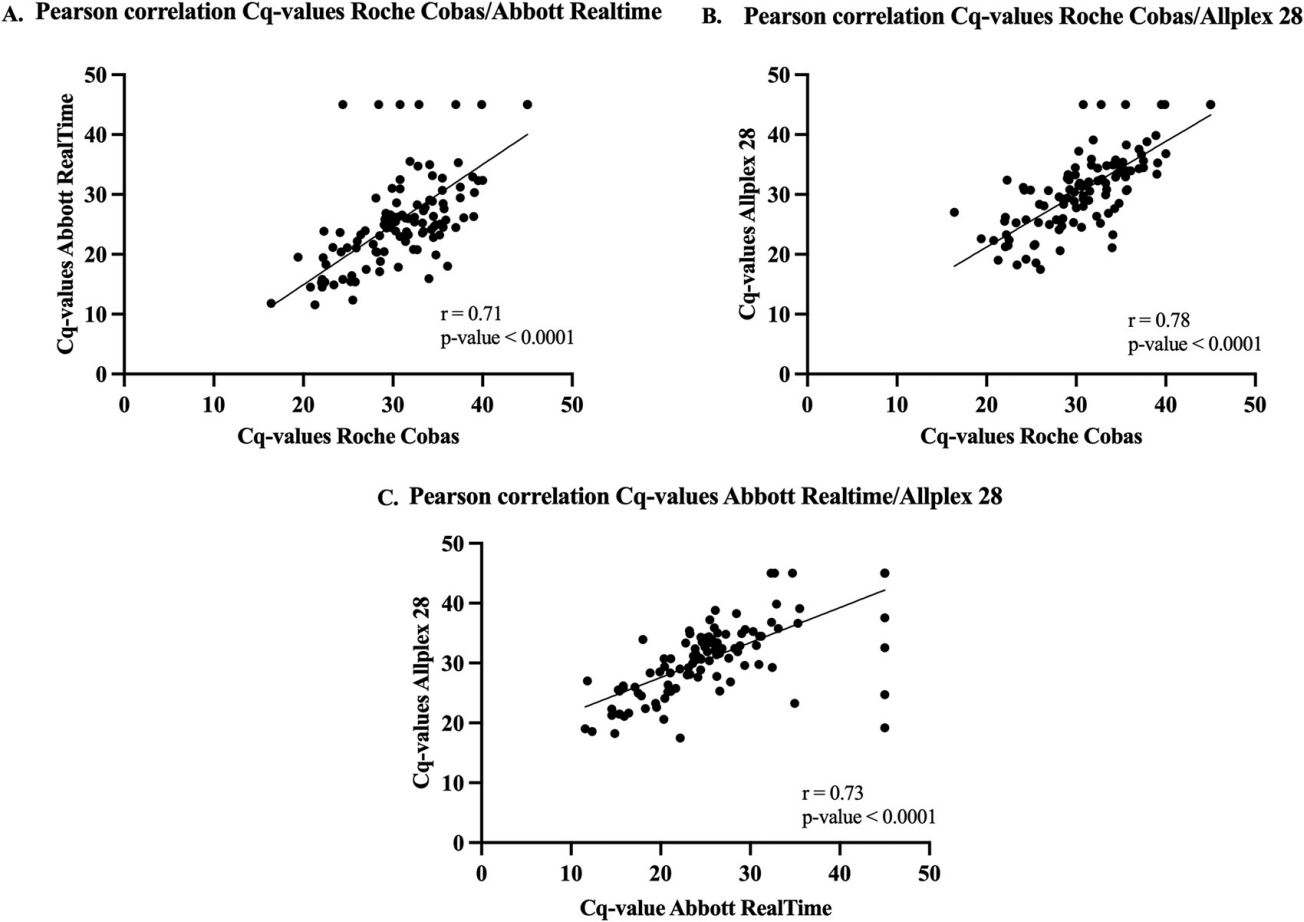
Correlation between the *C_q_* values of the Roche Cobas and Abbott RealTime HPV assays (A), the Roche Cobas and Allplex HPV28 assays (B), and the Abbott RealTime and Allplex HPV28 assays (C). Graphs visualize a significant Pearson’s correlation between the *C_q_* values of all qPCR HPV assays (*r* = 0.71, *P* < 0.001; *r* = 0.78, *P* < 0.001; 0.73, *P* < 0.001), indicating a strongly positive relationship.

Pearson correlations between the *C_q_* values of different HPV assays are shown in Fig. 1. All *C_q_* values of the different HPV assays were demonstrated to be strongly and positively correlated (r values of 0.71, 0.73, and 0.78) and statistically significant (all *P* values are <0.0001).

## DISCUSSION

In this study, we aimed to compare the performance of a new HPV assay, i.e., the Seegene Allplex HPV28 assay, and the Roche Cobas 4800 HPV, Abbott RealTime HR HPV, and Anyplex II HPV28 assays. Earlier studies evaluated and compared the Roche Cobas, Abbott RealTime, and Anyplex II HPV28 assays to each other (13, 14, 17) and to other HPV DNA assays (11, 15, 18, 19). However, to the best of our knowledge, this is the first study to compare the performances of these HPV assays with that of the novel Allplex HPV28 assay.

### Comparison between the included HPV assays using the manufacturer’s positivity cutoffs.

Overall, HPV DNA positivity rates within our study ranged between 78.9% and 92.9% (mean positivity rate of 87.3%), with the highest positivity rate detected for the Roche Cobas HPV assay. These findings are in line with previous research from Cho and colleagues (2019), who analyzed vaginal self-samples with the Abbott RealTime, Roche Cobas, and Anyplex II HPV28 assays and hereby also reported the highest positivity rate for the Roche Cobas assay (mean positivity rate of 87.4%) (20). Comparable to earlier research (14), the positivity rates of HPV DNA increased with higher cervical cytology grades for all included HPV assays. For the ASC-H cervical cytology, however, the Abbott RealTime, Anyplex, and Allplex HPV28 assays demonstrated deviating results, indicating reduced positivity rates compared with LSIL cytology. While the Roche Cobas assay detected all positive cases (*n* = 17) of ASC-H cervical cytology, the Anyplex and Allplex HPV28 assays both missed one case and the Abbott RealTime missed three.

Regarding the concordance among the four HPV assays, test results agreed in 85.9% of all cases when using the Abbott RealTime manufacturer’s *C_q_* cutoff for positivity (<32.00). Of all samples, 11 samples (9.7%) tested positive with all HPV assays, except for the Abbott RealTime assay. Slightly lower than the research of Park and colleagues (14), our results by the Roche Cobas and Abbott RealTime assay demonstrated an overall agreement of 85.9%. Further intercomparison showed a perfect (100.0%) agreement between the Anyplex and Allplex HPV28 assays and an agreement of 95.6% and 90.4% between the Seegene assays and the Roche Cobas and Abbott RealTime assays, respectively.

### Adaptation of qPCR HPV assay positivity cutoffs.

Importantly, the not detected but amplified (NDBA) phenomenon for the Abbott RealTime assay, i.e., the event in which the assay reports samples to be negative despite showing an amplification curve with a *C_q_* value higher than the manufacturer’s positivity cutoff (32.00), as described by earlier research of Kim and colleagues (21), also occurred frequently within our sample selection. Kim and colleagues (21) suggested that samples NDBA should be regarded as equivocal, not negative, considering that they could be a sign of a low-copy presence of hrHPV. Consequently, a comparison between the four HPV assays was also performed using a less stringent *C_q_* cutoff range for positivity on the Abbott RealTime assay, being 32.00 to 36.00. After analyzing the results using this adapted cutoff, the three ASC-H samples that were initially reported as negative nevertheless demonstrated a presence of hrHPV but with associated *C_q_* values of 32.93, 35.32, and 32.32. Moreover, it could be noted that the ASC-H sample that was missed with both Seegene tests was one of the three ASC-H samples that was also missed by the Abbott RealTime assay, indicating that hrHPV DNA might be present in this sample but with low viral loads. For the two HSIL cases initially missed by the Abbott RealTime assay, a similar situation could be observed (*C_q_*, ≥32.00), with results reported as negative indicating *C_q_* values of 32.37 and 32.47. Of the 11 samples that initially showed discrepancies for the Abbott RealTime assay, 7 (63.6%) could be explained by a similar phenomenon as the one explained above (*C_q_* value, ≥32.00). For the four remaining discordances, as well as for the five samples testing positive with the Roche Cobas assay but negative with all other HPV assays, no NDBA cases were observed. However, as shown by multiple previous studies, the Roche Cobas 4800 assay is known for having a relatively high sensitivity compared with other HPV assays (12, 13, 17). When NDBA samples were regarded as positive, the overall agreement between the four HPV assays moreover increased to 91.2%. Additionally, the agreement between the Abbott RealTime and the two Seegene assays increased to 96.5%, while the agreement between the Abbott RealTime and Roche Cobas assays rose to 92.9%, which is a result comparable to the one of Park and colleagues (14). Overall, our analysis hereby demonstrated considerable changes when comparing the results both before and after adjustment of the Abbott RealTime *C_q_* cutoff for positivity. As such, our study supports the assumption of other previous studies which concluded that the, often for specific circumstances validated and optimized, manufacturer’s *C_q_* cutoffs for positivity in HPV assays should always be handled with caution, especially when performing research within different age categories (23), socioeconomic settings (24), or types of (self-)sampling (25).

### Remarks on the included HPV assays and limitations of the study.

According to our experience, the novel Allplex HPV28 assay had a user-friendly and automated workflow with a short hands-on time, had an open platform which facilitates the use of add-on assays, and provided quick and easy-to-interpret results. Notably, our study included two assays that display individual results for HPV16 and HPV18, along with a pooled result for the other 12 hrHPV genotypes (Roche Cobas and Abbott RealTime) and two assays that are able to detect and differentiate all 14 distinct hrHPV genotypes (Anyplex and Allplex HPV28). Consequently, it should be remarked that results found within our study refer to the agreement in the presence of hrHPV genotypes in general (HPV16, HPV18, and other hrHPVs), rather than of specific genotypes. Considering that discrepancies within specific hrHPV genotypes could still occur without being displayed in our test results, as demonstrated in Table S1 for the Anyplex and Allplex HPV 28, we therefore recommend future studies to include extended genotyping techniques to strengthen the precision of assay comparison. This recommendation could be of particular interest for the further implementation of extended hrHPV genotyping in clinical settings, which may aid in the improved risk assessment of cervical lesions in those with abnormal cytology and associated referral for colposcopy. Lastly, due to the unavailability of the Hologic ThinPrep medium in Ecuador, brushes collected here were instead rinsed in Roche Cell Collection medium. As this origin-specific use of preservation medium results in a minimal chance of performance bias because of potential increased or decreased compatibility with a specific HPV assay, we acknowledge this as a limitation of our study.

### Conclusion.

Our study proved that the novel Allplex HPV28 assay has a good diagnostic performance in comparison with the well-known, validated, and frequently used Roche Cobas, Abbott RealTime, and Anyplex II HPV28 assays. Together with its ability to detect and quantify 28 HPV genotypes, the Allplex HPV28 assay could therefore potentially provide great opportunities for the simplification and standardization of future diagnostic testing programs. Nevertheless, further research on the safety, efficiency, and cost-effectiveness of newly developed HPV DNA assays for testing on self-collected specimens remains essential to support their implementation in practice and thereby improve future cervical cancer screening programs in all settings.

## MATERIALS AND METHODS

### Study design and population.

The current cross-sectional study was part of the ELEVATE project (https://elevate-hpv.com), an acronym for “EarLy dEtection of cerVical cAncer in hard-to-reach populations of women through portable and point-of-care HPV TEsting.” Within this project, a prospective collection of cervicovaginal samples (liquid-based cytology) from women was performed at the University Hospital of Ghent (Belgium), Instituto Portugues de Oncologia de Lisboa (Portugal), and Barretos Cancer Hospital (Brazil) and the Sociedad de Lucha contra el Càncer (SOLCA), Vicente corral Moscoso, Asociacion Pro Bienestar De La Familia Ecuatoriana (APROFE), and Jose Carrasco Hospitals (Ecuador).

### Recruitment procedures.

The recruitment of participants with either cervical lesions or normal cytology was performed at the gynecological outpatient centers in each location. Women were eligible for inclusion if (i) they were aged 25 to 65 years, (ii) they were sexually active and not pregnant, (iii) had no history of hysterectomy, and (iv) had never been treated for cervical lesions or cancer. Women were excluded from the study if they (i) were menstruating at the time of their visit or (ii) had sexual intercourse with vaginal penetration within the last 48 h before their visit. Study participants were informed on the study goals and procedures by their attending gynecologist, and all participants provided written informed consent for the use of their samples for HPV DNA detection. Participants were not financially rewarded.

### Sample collection and selection.

From each of the included ELEVATE participants, two mocked self-samples were collected by a gynecologist. To achieve this collection, the gynecologists held two Viba-Brush self-testing devices (Rovers, Oss, The Netherlands) together for sampling and attempted on mimicking self-collected specimens by swabbing over and around the cervix rather than going endocervical and thereby also touching a part of the more superficial vaginal canal. In Belgium, Portugal, and Brazil, the brushes were each rinsed in Hologic ThinPrep pap test medium (Hologic Inc., Mississauga, ON, Canada). Due to the unavailability of Hologic ThinPrep pap test medium in Ecuador, the brushes here were rinsed in Roche cell collection medium (Roche Diagnostics, Indianapolis, IN). Hereafter, one of the samples was sent to Barretos Cancer Hospital, Brazil, for analysis via the Roche Cobas HPV assay and the second one of the pair was sent to Ghent University Hospital, Belgium, for further HPV testing. From this ELEVATE cohort, a selection of 114 participants, from whom cervical cytology data were available, was made for the current study. This selection included a balanced representation of the different sampling locations (Belgium, Brazil, Ecuador, and Portugal) and cervical cytology categories (normal cytology, LSIL, HSIL, ASC-US, ASC-H, and cervical cancer). On this selection, additional HPV testing via the Abbott RealTime HR HPV assay, the Anyplex II HPV28 detection assay, and the Allplex HPV28 detection assay was performed at Ghent University Hospital, Belgium. Samples were stored at an optimum temperature of 2 to 8°C in between HPV testing.

### qPCR HPV assays.

The samples were analyzed using four qPCR HPV assays, as follows: (i) the Abbott RealTime HR HPV assay (Abbott Molecular Inc., Des Plaines, IL), (ii) the Roche Cobas 4800 HPV assay (Roche Diagnostics, Indianapolis, IN), (iii) the Anyplex II HPV28 detection assay (Seegene Inc., Seoul, South Korea), and (iv) the Allplex HPV28 detection assay (Seegene Inc.). All four assays use a combination of multiple primers and probes to amplify and detect a conserved L1 region of different HPV genotypes (Table 1). Moreover, to verify the presence of human cells, the assays all target the human β-globin gene as internal quality control (IQC). The Abbott RealTime HR HPV assay was carried out using an m2000rt automated analyzer (Abbott Molecular Inc.) for PCR amplification and detection, using a *C_q_* value of 32.00 as the positivity cutoff for HPV16, HPV18, and other hrHPVs, after sample preparation and extraction on an m2000sp instrument (Abbott Molecular Inc.). The Roche Cobas HPV assay was performed on the Cobas 4800 system (Roche Diagnostics), using a *C_q_* cutoff value of 40.50 for HPV16 positivity and 40.00 for HPV18 and other hrHPV positivity. Both the Anyplex II HPV28 and Allplex HPV28 detection assays were performed using a CFX96 real-time thermocycler (Bio-Rad, Hercules, CA). In the Anyplex II HPV28 detection assay, the HPV load was categorized as either high (+++), medium (++), or low (+), if the *C_q_* value was ≤31.00, between 31.00 and 40.00, or ≥41.00, respectively; in the Allplex HPV28 detection assay, a *C_q_* cutoff value of 42.00 was used for positivity for all hrHPV genotypes. All assays and procedures (including the *C_q_* cutoffs) were executed following the manufacturers’ instructions. While the Abbott RealTime HR HPV and Roche Cobas HPV assay provide individual results for HPV16 and HPV18 along with a pooled result for the other 12 hrHPV genotypes, the Anyplex II HPV28 and Allplex HPV28 detection assays are able to simultaneously detect, differentiate, and quantify 28 distinct HPV genotypes, including low-risk HPV genotypes (Table 1). To enable a comparison between the four different assays, this study collected and included results only of hrHPV genotypes for the Anyplex II HPV28 and Allplex HPV28 detection assays.

### Data analysis.

All results were statistically analyzed and visualized using the Prism 9 software (GraphPad Software, San Diego, CA). Per HPV assay, the HPV DNA-positive cases and cases of coinfection were determined for different cervical cytology types. Notwithstanding that both the Anyplex and Allplex HPV28 assays can detect the 14 hrHPV genotypes individually, the Roche Cobas and Abbott RealTime assays can only distinguish HPV16, HPV18, and other hrHPV genotypes. Accordingly, to allow for a comparison, we clustered all hrHPV genotypes except for HPV16 and HPV18 within the Anyplex and Allplex HPV28 results as well. Consequently, samples were considered coinfected only when they were infected with HPV16 and/or HPV18 and at least one other hrHPV genotype. Coinfection rates were calculated by dividing the number of coinfected cases by the number of cases positive for HPV DNA. As no reference standard assay for the use of mocked self-samples was available, concordance in terms of detecting and genotyping (HPV16, HPV18, and other hrHPV) HPV among the four different HPV assays was assessed by the positive percent agreement, the negative percent agreement, and the overall percent agreement with Cohen’s kappa score calculation. Within these head-to-head comparisons, either the Roche Cobas 4800 assay or the Abbott RealTime HR HPV assay was used as the nonreference standard. Two assays were negatively agreeing if they both demonstrated a negative result for all hrHPV genotypes, were positively agreeing if both detected at least one hrHPV genotype, and were overall agreeing if they both generated the same result (HPV16/HPV18/other hrHPV genotypes). A kappa statistic (κ) of 0.81 to 1.00 was interpreted as an almost perfect agreement, 0.61 to 0.80 as a good agreement, 0.41 to 0.60 as a moderate agreement, 0.21 to 0.40 as a fair agreement, 0.00 to 0.20 as a minimal agreement, and < 0.00 as a poor agreement between the different tests (22). For all HPV assays, concordance results were analyzed based on the manufacturer’s recommended *C_q_* cutoff for positivity. However, as described in earlier research (21), the Abbott RealTime assay often reports samples as HPV negative, while showing an amplification curve with a *C_q_* value above the manufacturer-defined cutoff for positivity (<32.00) (NDBA [not detected but amplified]). Therefore, in the current study, concordance and agreement between the different HPV assays was determined both by using the Abbott RealTime manufacturer’s recommended *C_q_* cutoff for positivity (<32.00) and by using a less stringent *C_q_* range for positivity on NDBA samples (32.00 to 36.00). To examine the correlation between the HPV loads detected by different HPV assays (except for the semiquantitative Anyplex II HPV28), Pearson correlation coefficients for the *C_q_* values were calculated. For negative samples, an arbitrary *C_q_* value of 45.0 was used. *P* values of less than 0.05 were considered to be statistically significant.

### Ethical aspects.

Ethical approval was obtained by the ethical committee of Ghent University Hospital (reference 2019/1687), the institutional review board (IRB) committee of Cuenca University (reference 2018-074EO-I-Ext#1), the ethical committee for Health of Instituto Portugues de Oncologia de Lisboa Francisco Gentil (reference UIC/1267 and 290/021), and the National Research Ethics Commission of Brazil (reference 16983119.7.0000.5437). Samples were collected in accordance with guidelines and regulations of the Helsinki Declaration, and all data were handled according to General Data Protection Regulations (GDPR).
