# Supplementary material for: Comparison between the Roche Cobas 4800 Human Papillomavirus (HPV), Abbott RealTime High-Risk HPV, Seegene Anyplex II HPV28, and Novel Seegene Allplex HPV28 Assays for High-Risk HPV Detection and Genotyping in Mocked Self-Samples
Source: Microbiol Spectr. 2023 Jun 7;11(4):e00081-23. doi: 10.1128/spectrum.00081-23 (PMC10433804; doi:10.1128/spectrum.00081-23)
Supplement: Supplemental file 1 — Table S1. Download spectrum.00081-23-s0001.docx, DOCX file, 0.01 MB [file spectrum.00081-23-s0001.docx]

| **Supplementary Table 1.** Discrepancies between the Anyplex^TM^ and Allplex^TM^ II HPV28 assay per hrHPV genotype^a^. | | | |
| --- | --- | --- | --- |
|  | HPV DNA positive cases (n) | |  |
| HR HPV genotype | AnyplexTM 28 | AllplexTM 28 | Agreement (%) |
| HPV16 (n = 13) | 13 | 13 | 100.0 |
| HPV18 (n = 8) | 8 | 8 | 100.0 |
| HPV31 (n = 14) | 14 | 14 | 100.0 |
| HPV33 (n = 6) | 6 | 6 | 100.0 |
| HPV35 (n = 8) | 8 | 8 | 100.0 |
| HPV39 (n = 8) | 8 | 8 | 100.0 |
| HPV45 (n = 8) | 8 | 8 | 100.0 |
| HPV51 (n = 10) | 9 | 10 | 90.0 |
| HPV52 (n = 14) | 13 | 14 | 92.6 |
| HPV56 (n = 11) | 11 | 11 | 100.0 |
| HPV58 (n = 13) | 13 | 13 | 100.0 |
| HPV59 (n = 12) | 10 | 12 | 83.3 |
| HPV66 (n = 10) | 10 | 10 | 100.0 |
| HPV68 (n = 6) | 6 | 6 | 100.0 |
| *^a^Abbreviations: HR, high-risk.* | | |  |
